# Dosing Regimens of Immune Checkpoint Inhibitors: Attempts at Lower Dose, Less Frequency, Shorter Course

**DOI:** 10.3389/fonc.2022.906251

**Published:** 2022-06-20

**Authors:** Mengjie Jiang, Yujie Hu, Gang Lin, Chao Chen

**Affiliations:** Department of Radiotherapy, The First Affiliated Hospital of Zhejiang Chinese Medical University, Zhejiang Provincial Hospital of Traditional Chinese Medicine, Hangzhou, China

**Keywords:** immune checkpoint inhibitors, adverse effects, optimization dosing regimens, lower dosage, selectively discontinuation

## Abstract

Immune checkpoint inhibitors (ICIs) are a revolutionary breakthrough in the field of cancer by modulating patient’s own immune system to exert anti-tumor effects. The clinical application of ICIs is still in its infancy, and their dosing regimens need to be continuously adjusted. Pharmacokinetic/pharmacodynamic studies showed a significant plateau in the exposure-response curve, with high receptor occupancy and plasma concentrations achieved at low dose levels. Coupled with concerns about drug toxicity and heavy economic costs, there has been an ongoing quest to reevaluate the current ICI dosing regimens while preserving maximum clinical efficacy. Many clinical data showed remarkable anticancer effects with ICIs at the doses far below the approved regimens, indicating the possibility of dose reduction. Our review attempts to summarize the clinical evidence for ICIs regimens with lower-dose, less-frequency, shorter-course, and provide clues for further ICIs regimen optimization.

## 1 Introduction

Immune checkpoint inhibitors (ICIs) are revolutionary breakthroughs in the field of cancer in recent years, which have changed the traditional treatment paradigm. At present, the relatively proven ICIs in clinical application include cytotoxic T-lymphocyte-associated protein 4 (CTLA-4) inhibitors and programmed cell death protein-1 (PD-1) receptor inhibitors/programmed cell death ligand 1 (PD-L1) inhibitors. CTLA-4 is a transmembrane receptor on T cells, which can compete with CD28 to prevent co-stimulation and induce T cell cycle arrest. CTLA-4 inhibitors block the above process and restore the function of T cells to eradicate tumor cells. The U.S. Food and Drug Administration (FDA) approved CTLA-4 inhibitors include: ipilimumab, tremelimumab. PD-1 is expressed on tumor-infiltrating lymphocytes (mainly CD4+ T cells), B cells, natural killer cells, monocytes and dendritic cells, while PD-L1 is highly expressed on tumor cells. The binding of PD-1 to PD-L1 mediates a co-inhibitory signal of T cell activation, thus leading to tumor immune escape. PD-1/PD-L1 inhibitors block the PD-1 signaling pathway, partially restoring T-cells recognition of tumors and inducing immune normalization. Currently FDA-approved PD-1 inhibitors include: nivolumab, pembrolizumab, cemiplimab, dostarlimab, and PD-L1 inhibitors include: atezolizumab, avelumab, durvalumab. In fact, most research on dose intensity reduction of ICI focused on nivolumab, pembrolizumab and ipilimumab, our review also mainly focused on these three ICIs.

ICIs belong to monoclonal antibody and their pharmacokinetic/pharmacodynamic properties are distinctly different from those of traditional cytotoxic and small molecule drugs; therefore, determining the optimal dose of ICIs using traditional drug models may face many difficulties. The recommended dosing regimens for ICIs have evolved as experience accumulates. ICIs were initially administered based on body weight, and as population pharmacokinetic data accumulated, fixed-dose regimen was found to improve convenience and reduce waste while preserving efficacy, thus FDA approved nivolumab 240 mg Q2W equivalent to 3 mg/kg Q2W and pembrolizumab 200 mg Q3W equivalent to 2 mg/kg Q3W. Subsequently, high-dose, extended-interval dosing regimens (e.g. nivolumab 480 mg Q4W and pembrolizumab 400 mg Q6W) were added to all approved adult indications based on silico simulations (1–4), and validated in prospective clinical trials (5–7). Notably, the choice of average body weight is not consistent across ICIs: 240mg fixed dose of nivolumab is numerically equivalent to 3mg/kg dose for 80kg patients, while 200mg pembrolizumab corresponds to 2mg/kg for 100kg patients and 750mg durvalumab corresponds to 10mg/kg for 75kg patients. Cancer patients are often combined with cachexia resulting in underweight. The average weight of patients using ICI is about 75 kg (8–10), and the Asian population tend to have lower weight. In clinical practice, clinicians may reduce the dose or delay the administration of ICIs concerns about the patient’s physical condition or adverse effects of drugs, as well as for economic reasons or patient requests, but significant survival benefits can still be seen, which may provide clues to optimize the dosing regimens. In terms of duration of therapy, the majority of ICIs are given for one year as adjuvant or consolidation therapy, which is entirely in reference to the duration of adjuvant chemotherapy, and there is no evidence to compare longer or shorter courses. For patients with advanced/metastatic tumors, treatment usually lasts two years or until disease progression or unacceptable toxicity occurs. For those patients with durable stability, there is no definitive answer as to when to discontinue ICIs therapy.

Considering the economic and convenience reasons, many scholars suggest the need to reevaluate the current ICI dosing regimens. They proposed the concept of interventional pharmacoeconomics, hoping to reduce health care costs and perhaps also adverse effects while maintaining treatment efficacy through the development of new dosing regimens (11, 12). The main strategies include lower doses, less frequent dosing, shorter duration of treatment and therapeutic substitution, which have successfully improved the clinical practice of many drugs (e.g., abiraterone, ibrutinib, trastuzumab) (11, 12), also provide opportunities for dose reduction of ICIs (13, 14). In this review, we mainly discuss the issues of dosing intensity reduction and treatment duration selection, and the administration strategy of ICIs in the context of the COVID-19 pandemic.

## 2 ICIs-Related Adverse Effects

The incidence of irAEs is 70-90% for any grade, 10-40% for grade 3/4, and 0.3-1.2% for fatal (grade 5) irAEs (15–22). An increased incidence and grade of irAEs as well as earlier onset could be observed in combination therapy (22). irAEs can occur in almost all organs throughout the body. Cutaneous toxicity is one of the most common irAEs, occurring in 1/3-1/2 of patients treated with ICIs, manifesting primarily as rash, pruritus, vitiligo (22, 23). The incidence of endocrine toxicity is 40% (16), mainly affecting thyroid, pituitary, and islet functions, requiring regular monitoring of hormone levels and timely hormone replacement therapy. Diarrhea is also a common side effect with an incidence of 15-45% (24). Colitis is the most common type of high-grade irAEs and one of the leading causes of discontinuation (22, 25). Immune pneumonitis is relatively rare but potentially fatal, with an incidence of 3-5% in clinical trials, and appears to be more common (9-19%) in real-world studies, among which grade 3-4 pneumonitis accounting for 30-50% of cases, and 10% patients may develop an infection that leads to death (22, 26–30). Other rare irAEs include hematologic toxicity, nephrotoxicity, immune hepatitis, immune myocarditis, neurological irAEs, etc. Distinguish from cytotoxic drugs, ICIs are generally administered continuously for a long time, chronic toxicity (even low-grade toxicity) is likely to be intolerable for patients. Approaches to reduce irAEs include more precise selection of targeted population, optimization of drug regimens, whole course management, and prophylactic application for high-risk patients.

## 3 Dosing Regimen Optimization

Dose optimization studies on ICIs are limited, focusing on nivolumab, pembrolizumab, and ipilimumab. An extensive literature search was conducted for the three most widely used ICIs to collect clinical data of each drug. Retrieval method: Firstly, in the PubMed database, the literature was searched by “Clinical Trial”, “Prospective Studies”, “Retrospective Studies” and “nivolumab”, “pembrolizumab”, “ipilimumab”. Secondly, search clinical trials of each drug in Clinicaltrials.gov. Inclusion criteria: retrospective and prospective studies of three ICIs that include off-label dosing regimens, whether monotherapy or combined therapy, whether directly comparing different dosing regimens or simply including cohorts with off-label dosing regimens. Although most attempts at off-labelled dosing regimens are pharmacokinetic/pharmacodynamic simulations, subgroup data from early clinical trials or retrospective studies with small samples, they can still provide clues to the optimization of ICIs (**Table 1**).

**Table 1:** Retrospective and Prospective Studies of Dose Reduction of ICIs.

ICIs	Study	Author (Year)	Object	No. of Patients	Dosing Regimen	Median Follow-Up (months)	ORR	Median PFS (months)	Median OS (months)	All grade irAEs (Grade 3-4 irAEs) %
**NIVO**	CA209-003 (NCT00730639)	Topalian (31) (2014)	Advanced melanoma	107			33/107 (30.8%)	3.7	16.8	84.1 (22.4)
				17	0.1 mg/kg q2w		6/17 (35.3%)	3.6	16.2	76.5 (29.4)
				18	0.3 mg/kg q2w		5/18 (27.8%)	1.9	12.5	77.8 (16.7)
				35	1 mg/kg q2w		11/35 (31.4%)	9.1	25.3	97.1 (14.3)
				17	3 mg/kg q2w		7/17 (41.2%)	9.7	20.3	88.2 (35.3)
				20	10 mg/kg q2w		4/20 (20.0%)	3.7	11.7	70.0 (25.0)
	NCT01176461	Weber (32) (2013)	Unresectable stage III or IV melanoma	34		20.3				
				10	1 mg/kg q2w		3/10 (30.0%)			
				13	3 mg/kg q2w		4/13 (30.7%)			
				11	10 mg/kg q2w		1/11 (9.1%)			
	Checkmate 010 (NCT01354431)	Motzer (33) (2015)	Stage IV RCC	168		≥24				
				60	0.3 mg/kg q2w		12/60 (20.0%)	2.7	18.2	75 (5)
				54	2 mg/kg q2w		12/54 (22.2%)	4.0	25.5	67 (17)
				54	10 mg/kg q2w		11/54 (20.4%)	4.2	24.7	78 (13)
	Retrospective study	Yoo (34) (2018)	Stage IIIB/IV or recurrent NSCLC	47		5.2	7/47 (14.9%)	1.1	12.5	
				18	Low-dose group (20/100 mg q3w)	4.7	3/18 (16.7%)	3.0	12.5	
				29	Standard-dose group (3 mg/kg q2w)	5.6	4/29 (13.8%)	1.0	8.2	
	Retrospective study	Zhao (35) (2021)	Advanced RCC	32			15/32 (46.9%)	6.0	10.0	40.6 (15.6)
				16	Low-dose group (<2.15 mg/kg)		7/16 (43.8%)	7.0	NR	50 (18.8)
				16	High-dose group (> 2.15 mg/kg)		8/16 (50.0%)	7.0	28.0	31.3 (12.5)
	NCT03343665	Lepik (36) (2020)	Relapsed/refractory Hodgkin Lymphoma	30	40 mg q2w	19.2	21/30 (70.0%)	18.4	NR	93.3 (13.3)
	NCT02985554	Wang (37) (2020)	Post-alloHCT without relapse	4	1 mg/kg q2w (The study was terminated early due to serious side effects)					100 (50)
	CTEP 9204 (NCT01822509)	Davids (38) (2020)	Relapsed hematologic malignancies after alloHCT	28		11	8/28 (29%)	3.7	21.4	
				6	1 mg/kg q2w		3/6 (50%)			2 DLT
				22	0.5mg/kg q2w		5/22 (22.7%)			4 DLT
**PEMB**	Retrospective study	Low (39) (2021)	Advanced NSCLC	114		14.8				
				65	100 mg q3w		21/46^a^ (45.7%)	6.8	14.3	(17)
				49	200 mg q3w		17/42^a^ (40.5%)	4.2	19.8	(22)
	Retrospective study	Sehgal (64 ) (2021)	Advanced NSCLC	92			55/92 (59.8%)			59 (30)
				27	Extended-dose group (≥ 2 cycles at intervals > 3 weeks + 3 days)		18/27 (66.7%)	23.3	NR	70 (26)
				65	Standard-dose group (all cycles every 3 weeks or 1 cycle > 3 weeks + 3 days)		37/65 (56.9%)	7.0	15.4	54 (32)
	Retrospective study	Chen (40) (2020)	Relapsed/refractory Hodgkin lymphoma	11	100 mg q3w		11/11 (100%)	35	NR	27.3
**NIVO+IPI**	CheckMate 032 (NCT01928394)	Antonia (41) (2016)	Recurrent SCLC							
				61	NIVO1+IPI3^b^	12.0	14/61 (23.0%)	2.6	7.7	79 (30)
				54	NIVO3+IPI1^c^	8.7	10/54 (18.5%)	1.4	6.0	75 (19)
	CheckMate 032 (NCT01928394)	Sharma (42) (2019)	Locally advanced or metastatic urothelial carcinoma							
				92	NIVO1+IPI3^b^	≥7.9	35/92 (38.0%)	4.9	15.3	80.4 (39.1)
				104	NIVO3+IPI1^c^	≥38.8	28/104 (26.9%)	2.6	7.4	84.6 (30.8)
	CheckMate 032 (NCT01928394)	Janjigian (43) (2018)	Metastatic esophagogastric cancer							
				49	NIVO1+IPI3^b^	24	12/49 (24.4%)	1.4	6.9	84 (47)
				52	NIVO3+IPI1^c^	22	4/52 (7.7%)	1.6	4.8	75 (27)
	CheckMate 511 (NCT02714218)	Lebbé (44) (2019)	Advanced melanoma							
				178	NIVO1+IPI3^b^	18.6	90/178 (50.6%)	8.9	NR	93.8 (48.3)
				180	NIVO3+IPI1^c^	18.8	82/180 (45.6%)	9.9	NR	85.6 (33.9)
	OpACIN-neo (NCT02977052)	Rozeman (45) (2019)	Neoadjuvant stage III melanoma			8.3				
				30	NIVO1+IPI3^d^		24/30 (80.0%)	NR		97 (40)
				30	NIVO3+IPI1^e^		23/30 (76.7%)	NR		97 (20)
	CheckMate 040 (NCT01658878)	Yau (46, 47) (2020)	Advanced hepatocellular carcinoma			30.7				
				50	NIVO1+IPI3^b^		16/50 (32.0%)		22.8	94 (53)
				49	NIVO3+IPI1^c^		13/49 (26.5%)		12.5	71 (29)
				49	NIVO (3 mg/kg q2w) + IPI (1 mg/kg q6w)		14/49 (28.6%)		12.7	79 (31)
	NCT03222076	Kaseb (48) (2022)	Perioperative hepatocellular carcinoma	14	Preoperative: NIVO (240 mg q2w * 3 doses) + IPI (1 mg/kg * 1 dose) Postoperative: NIVO (480mg q4w) + (IPI 1 mg/kg q6w)		3/11 (27.3%)	19.53		(43)
	CheckMate 142 (NCT02060188)	Overman (49) (2018)	MSI-H/dMMR mCRC^f^	119	NIVO3+IPI1^c^	13.4	65/119 (54.6%)	NR	NR	73 (32)
	CheckMate 142 (NCT02060188)	Lenz (50) (2022)	MSI-H/dMMR mCRC^f^	45	NIVO (3 mg/kg q2w) + IPI (1 mg/kg q6w)	29.0	31/45 (68.9%)	NR	NR	(22)
	CheckMate 920 (NCT02982954)	Unpublished (51)	Advanced non-clear cell renal cell carcinoma							
				106	NIVO 6 mg/kg + IPI 1 mg/kg q8w, alternating with NIVO 480 mg q8w; the altered dosing was staggered every 4 weeks		33/96 (34.4%)	4.8	NR	54 (24)
	CheckMate 920 (NCT02982954)	Tykodi (52) (2022)	Advanced non-clear cell renal cell carcinoma	52	NIVO3+IPI1^c^	19.0	9/46 (19.6%)	3.7	21.2	69 (23)
	MAYA trial (NCT03832621)	Morano (2022) (53)	MSS, MGMT silenced mCRC^g^	33	TMZ followed by IPI 1 mg/kg q8w+NIVO 480 mg q4w	23.1	6/33 (18.2%)^h^	7.1	18.4	91 (21)^h^
	CheckMate 012 (NCT01454102)	Hellmann (54) (2017)	Advanced NSCLC							
				38	NIVO (3 mg/kg q2w) + IPI (1 mg/kg q12w)	12.8	18/38 (47.4%)	8.1	NC^i^	82 (37)
				39	NIVO (3 mg/kg q2w) + IPI (1 mg/kg q6w)	11.8	15/39 (38.5%)	3.9	NC^i^	71 (33)
	Retrospective study	Kleef (55) (2021)	Unselected stage IV solid cancer patients	131	Interleukin-2 + NIVO (0.5 mg/kg) + IPI (0.3 mg/kg)	60	31.30%	7.1	19.3	48.1 (8.4)
	NCT02941744	Schwarze (56) (2022)	Adjuvant therapy following the resection of melanoma metastases							
				34	IPI 50 mg (1 dose) + NIVO 10 mg q2w (up to 4 does)	54.8	NA	19.8	NR	79 (9)
				21	NIVO 10 mg q2w *9 doses followed by NIVO 10 mg q8w *4 doses	44.3	NA	NR	NR	86 (5)
**PEMB+IPI**	KEYNOTE-029 (NCT02089685)	Carlino (57) (2020)	Advanced melanoma	153	PEMB2 + IPI1^j^	36.8	95/153 (62.1%)	NR	NR	96.1 (47.1)
		Long (58) (2021)		51	PEMB 200 mg q3w+ IPI 50 mg q6w	16.3	28/51 (54.9%)	NR	NR	100 (24)
				51	PEMB 200 mg q3w+ IPI 100 mg q12w	16.4	31/51 (60.8%)	NR	NR	96 (39)
	NCT02743819	Olson (59) (2021)	Anti-PD-1/L1 failure advanced melanoma	70	PEMB2 + IPI1^j^	12.0	20/70 (28.6%)	5	24.7	87 (27)
	KEYNOTE-021 (NCT02039674)	Gubens (60) (2019)	Later-line advanced NSCLC	44	PEMB2 + IPI1^j^	11.3	13/44 (29.5%)	4.1	10.9	64 (29)

Retrospective and prospective studies that include off-labeled dose de-escalation dosing regimens, whether monotherapy or combined therapy, whether directly comparing different dosing regimens or simply including cohorts with dose-reduction dosing regimens.

ICIs, immune checkpoint inhibitors; irAEs, ICIs-related adverse effects; ORR, objective response rates (CR + PR); PFS, progression-free survival; OS, overall survival; NIVO, nivolumab; PEMB, pembrolizumab; IPI, ipilimumab; NR, not reached; NC, not calculated; NA, not available; TPS, tumor proportion score; RCC, renal cell carcinoma; NSCLC, non-small-cell lung cancer; SCLC, small-cell lung cancer; alloHCT, allogeneic hematopoietic cell transplantation; DLT, dose-limiting toxicity. *, multiple the number of cycles.

a. Response rates were calculated in 88 patients who received pembrolizumab as first-line treatment.

b. Combination treatment with nivolumab 1 mg/kg q3w plus ipilimumab 3 mg/kg q3w for four cycles followed by nivolumab monotherapy.

c. Combination treatment with nivolumab 3 mg/kg q3w plus ipilimumab 1 mg/kg q3w for four cycles followed by nivolumab monotherapy.

d. Two cycles of ipilimumab 3 mg/kg q3w plus nivolumab 1 mg/kg q3w.

e. Two cycles of ipilimumab 1 mg/kg q3w plus nivolumab 3 mg/kg q3w.

f. Microsatellite instability-high/mismatch repair-deficient (MSI-H/dMMR) metastatic colorectal cancer (mCRC).

g. Microsatellite-stable (MSS) and O6-methylguanine-DNA methyltransferase (MGMT)-silenced metastatic colorectal cancer (mCRC).

h. The ORR obtained in the second ICIs treatment part was 18%, the incidence of any grade and grade ≥ 3 irAEs was 91% and 21%, respectively.

i. Not calculated due to a large proportion of patients having been censored at the time of analysis.

j. Combination treatment with pembrolizumab 2 mg/kg/200mg q3w plus ipilimumab 1 mg/kg q3w for four cycles followed by pembrolizumab monotherapy.

### 3.1 Pharmacokinetics/Pharmacodynamics Attempts

For most ICIs (except ipilimumab), there is no clear relationship between dose and efficacy or safety. The dose-response and exposure-response curves showed an obvious plateau, implying that increasing doses do not contribute to tumor control and that lower-dose ICIs may produce the same effect (61–63). Agrawal et al. found that the exposure-response relationship reached a plateau with nivolumab doses ≥1 mg/kg in melanoma and renal cell carcinoma, suggesting that low-dose regimen could be tried in high-immunogenic tumors (64). Receptor occupancy is maximized at low dose level, e.g., peripheral PD-1 receptor occupancy is saturated with 0.3 mg/kg nivolumab (64, 65), greater than 90% with 0.5 mg/kg pembrolizumab, 4 mg/kg atezolizumab, 3 mg/kg avelumab (66–68), and the soluble PD-L1 receptor was completely suppressed when durvalumab ≥ 0.3 mg/kg (69). The trough concentration (Cmin) at the recommended dose is also much higher than the target concentration. For example, the Cmin of 3 mg/kg atezolizumab exceeds the target concentration of 6 μg/mL (68, 70), while the labelled dose has a Cmin (>100 μg/mL) nearly 20 times higher than the target concentration (4, 71).

In terms of dosing intervals, prolonging the interval still maintains the pharmacodynamic parameters at effective levels. Simulated administration of nivolumab at 240mg Q4W/480mg Q8W regimen and pembrolizumab at 200mg Q6W regimen revealed that serum drug concentrations remained above the minimum effective concentration in more than 95% of patients (72). The Canadian Agency of Drugs and Technologies in Health simulated dosing regimens of pembrolizumab 4 mg/kg Q6W in patients weighing 70, 100, and 150 kg, all with trough target engagement above 97% (73). Comparison of the standard regimen of atezolizumab with several extended interval regimens showed that the predicted efficacy and safety of 1680 mg Q8W/1200 mg Q6W was not inferior to the standard 1200 mg Q3W (74).

Pharmacokinetic studies have shown that many variables can influence the clearance of ICIs, such as: gender, race, weight, performance status, tumor volume, drug response, and albumin levels (62, 75, 76). Over the treatment, drug clearance decreases as the responders’ performance status improves and tumor burden decreases (62). Therefore, it remains to be investigated whether less-frequent or lower-dose regimens could be administered in subsequent cycles for those patients who achieved good outcomes.

### 3.2 Clinical Evidence

#### 3.2.1 Nivolumab

In the phase I CA209-003 trial (31), melanoma patients received 0.1, 0.3, 1 mg/kg nivolumab, and the objective response rates (ORR) were 35%, 28%, and 31%, respectively. Patients who did not respond to the lower dose remained unresponsive to the higher dose (31). In another phase I clinical trial of melanoma, the ORR of patients receiving 1, 3 mg/kg nivolumab was 30% and 31%, respectively (32). In the Checkmate 010 study in renal cancer, nivolumab was administered at doses of 0.3, 2, or 10 mg/kg with similar efficacy (33). All of the above early clinical trials suggested that nivolumab may be effective at low doses. A retrospective study from Korea compared the low-dose nivolumab group (20 mg/100 mg Q3W) with the standard-dose group (3 mg/kg Q2W) in non-small cell lung cancer (NSCLC) with no significant difference in ORR, progression-free survival (PFS), and overall survival (OS) (34). Results from another retrospective studies in Singapore also showed that low-dose (100 mg/140 mg) nivolumab did not reduce efficacy in renal cancers (35). In a single-arm, open-label phase II study conducted in Russia, the ORR for 40 mg Q2W nivolumab in relapsed/refractory Hodgkin lymphoma was 70%, with 13/30 (43.3%) achieving complete remission (CR) (36). The results of these clinical studies further confirmed the previous hypothesis that highly immunogenic tumors may be effective at low doses of ICIs.

Based on the results of the CheckMate 205 study (77), The FDA approved 3 mg/kg nivolumab for relapsed/refractory classic Hodgkin lymphoma after autologous hematopoietic cell transplantation (HCT), but did not recommend it for patients with allogeneic HCT (alloHCT), primarily due to the high risk of graft-versus-host disease (GVHD) (78–80). However, in retrospective studies and case reports, some physicians have also used low-dose nivolumab (0.3–1.5 mg/kg) in post-alloHCT patients with success (81–84). A clinical trial of 1 mg/kg Q2W nivolumab in the post-alloHCT population was terminated early due to serious side effects (37). In another study, nivolumab was started at 1 mg/kg and dose-limiting toxicity was observed in 2/6 patients, severe irAEs and fatal GVHD still occurred in 4/22 patients even after nivolumab was reduced to 0.5 mg/kg (38), indicating that the use of nivolumab in post-alloHCT patients requires more caution and further studies are needed.

#### 3.2.2 Pembrolizumab

A retrospective study of advanced NSCLC found no significant differences in PFS, OS, or high grade irAEs between pembrolizumab 100mg and 200mg groups, either alone or in combination with chemotherapy (39). Similar survival outcomes were found between the extended-interval (>3 weeks + 3 days) and standard-interval groups of pembrolizumab in NSCLC patients, suggesting that extended dosing intervals may be available for patients with stable disease (85). Several cases have been reported in which complete remission was achieved with low-dose pembrolizumab in relapsed/refractory Hodgkin’s lymphoma, and even re-treatment with low-dose pembrolizumab remained effective (86, 87). In a series of studies in lymphoma, the ORR of 100 mg pembrolizumab was 100%, indicating the efficacy and safety of low-dose pembrolizumab, especially in the low-weight Asian population (40, 88). In addition, it is also recommended to administrate low dose pembrolizumab (50-100 mg) for post-alloHCT lymphoma (89).

#### 3.2.3 Ipilimumab

Rationalized medication of ipilimumab is focused on combination therapy with PD-1 inhibitors. Various attempts have been made to reduce the dose in the combination, but the choice of which drug to reduce and by how much to preserve maximum efficacy while reducing toxicity is inconclusive. Given the dose-dependent toxicity of ipilimumab, we prefer to reduce the dose of ipilimumab in combination therapy.

##### 3.2.3.1 Nivolumab+ Ipilimumab

###### 3.2.3.1.1 N3I1 vs N1I3

Although the combination of 3mg/kg nivolumab with 3mg/kg ipilimumab provides a potential survival benefit, severe toxicity limits its use (90). Common modified combinations are N3I1 (3mg/kg nivolumab+1mg/kg ipilimumab) and N1I3 (1mg/kg nivolumab+3mg/kg ipilimumab). Currently, the FDA approved N1I3 for the treatment of melanoma and hepatocellular carcinoma (91), N3I1 for renal and colorectal cancer, and the nivolumab 360mg Q3W plus ipilimumab 1 mg/kg Q6W regimen is recommended for lung cancer and malignant pleural mesothelioma (92). However, there is much controversy over the FDA recommended dosing regimens. Some studies have concluded that the mode of N1I3 is more effective than N3I1, with an acceptable overall safety profile despite a slight increase in irAEs (41–43, 93). In melanoma, the CheckMate 511 and OpACIN-neo trials compared these two combinations and found that with similar efficacy, the grade 3-5 irAEs was significantly fewer in the N3I1 group, suggesting that the N3I1 combination maybe more appropriate for melanoma (44, 45). Although patients with recurrent/advanced hepatocellular carcinoma showed the greatest survival benefit with the N1I3 regimen (46, 47), nivolumab combined ipilimumab 1 mg/kg Q6W regimen also showed good results in perioperative treatment (48). Microsatellite instability-high/mismatch repair-deficient metastatic colorectal cancer patients treated with nivolumab every 2 weeks plus low-dose ipilimumab every 6 weeks achieved robust and durable benefit, seem fewer side effects than N3I1 regimen (49, 50). In conclusion, the optimal combination regimen of nivolumab plus ipilimumab is still inconclusive, which may be related to cancer type and disease stage.

###### 3.2.3.1.2 Extended Interval Ipilimumab

Many studies have further extended the interval of ipilimumab to 6 weeks (49, 50, 94–97) or even longer. In the CheckMate 920 study of renal cancer, Cohort 1 patients received 6 mg/kg Q8W nivolumab plus 1 mg/kg Q8W ipilimumab, alternating with nivolumab 480 mg Q8W, with an ORR of 34.4%, median PFS 4.8 months, which was not significantly different from the results of several other cohorts using N3I1 regimen followed by nivolumab 480 mg Q4W (51, 52). The MAYA study evaluated the efficacy of temozolomide followed by nivolumab 480 mg Q4W plus ipilimumab 1 mg/kg Q8W in microsatellite-stable, O6-methylguanine–DNA methyltransferase–silenced metastatic colorectal cancer, with an ORR of 45% to the whole treatment strategy, median PFS of 7.1 months and median OS of 18.4 months (53). In CheckMate 012, nivolumab combined with ipilimumab 1 mg/kg Q6W or Q12W showed no significant differences in efficacy or safety in NSCLC patients (54).

A much lower-dose therapy of nivolumab (0.5 mg/kg) plus ipilimumab (0.3 mg/kg) combined with interleukin 2 and hyperthermia treating 131 cases of multiple advanced cancers showed an ORR of 31.3%, with median PFS reached 10 months, and the incidence of grade 3-4 irAEs was only 8.4% (55). In a Belgian single-center non-randomized phase II clinical trial, both nivolumab and ipilimumab were administrated as adjuvant therapy to melanoma at very low doses, with ipilimumab 50 mg (1 dose) plus nivolumab 10 mg Q2W (up to 4 does), and survival benefit was similar to the standard regimen (56).

##### 3.2.3.2 Pembrolizumab + Ipilimumab

There are relatively few studies of the combination of pembrolizumab and ipilimumab. In the KEYNOTE-029 study, melanoma patients received pembrolizumab 2 mg/kg Q3W in combination with ipilimumab 1 mg/kg Q3W followed by pembrolizumab monotherapy had an ORR of 62% and a 3-year OS of 73% (57). Cohort C further compared pembrolizumab combined with two ipilimumab dosing regimens (50 mg Q6W vs. 100 mg Q12W) and found little difference in efficacy between the two groups, but the side effects were more severe in 100mg Q12W regimen (58). For melanoma patients after anti-PD-1/L1 failure, the ORR for pembrolizumab combined with low-dose ipilimumab (1mg/kg Q3W) was 29% (59).

In the KEYNOTE-021 study, receiving pembrolizumab plus low-dose ipilimumab in later-line treatment for NSCLC resulted in an ORR of 30% and grade 3-5 irAEs rate of 29% (60). However, in first-line treatment of NSCLC with PD-L1 tumor proportion score (TPS) ≥ 50%, the addition of ipilimumab to pembrolizumab did not improve the survival but did result in increased toxicity (98). This reminds us of the need to further enhance our patient selection and biomarker selection.

Given these published findings, there is immense potential to reduce the dose intensity of ICIs by applying available pharmacology or clinical data, aided by prospective interventional pharmacoeconomic trials. Given the fact that ICIs are packaged in fixed single vials and drug sharing is not agreed upon in most hospitals, single-dose reduction may not be cost effective. Alternatively, extend the frequency of administrations (with the minimum effective plasma concentration) could reduce costs, adverse events, and patient inconvenience, and is in line with the context of Covid-19 pandemic. In the opinion of Goldstein et.al., the simplest approach to reduce the dose intensity of Atezolizumab or Nivolumab was using a standard dose with an extended interval far greater than the labeled regimens (13, 14). The determination of the specific dosing frequency needs to be further investigated and can also be considered with the help of Therapeutic Drug Monitoring (TDM) to more accurately guide the individualized dosing frequency. This concept of reduced dosing frequency could be first applied to ICIs given at fixed dose as monotherapies rather than in combination ICIs therapy with doses based on body weight.

## 4 Optimal Durations of ICIs

The treatment efficacy with different durations have been compared across several published clinical trials. Patients in the CA209-003 study were treated with nivolumab for up to 2 years with a 5-year OS of 16% (99), the CheckMate017/CheckMate057 study continued treatment until disease progression or intolerable toxicity with a 5-year OS of 13.4% (100); In the KEYNOTE-010 study in which treatment with pembrolizumab was administered for up to 2 years, 5-year OS was 15.6% (101), and the KEYNOTE-001 study required treatment until progression or intolerance, with a 5-year OS of 15.5% (102). Although inclusion criteria were not uniform across clinical trials, a long course of treatment does not necessarily mean long-term survival. Responders who discontinued due to irAEs had a similar survival benefit compared to those who completed the established course, implying that early discontinuation in these patients did not affect outcomes (103–105). Even more, it has been hypothesized that discontinuation of treatment due to severe irAE could itself serve as a biomarker of strong immune response and thus predict efficacy (103, 105, 106). While there is much evidence that short courses may provide durable benefits, this is not yet consistently standard practice worldwide currently. The National Comprehensive Cancer Network (NCCN)/the European Society for Medical Oncology (ESMO) guidelines generally recommend that, the duration of ICIs given as adjuvant or consolidation therapy was one year, and two years or until disease progression or unacceptable toxicity occurs for patients with later stage (107–111). Depth of response may play an important role in the determine of optimal treatment duration, and further exploration of prognostic biomarkers across different tumor types is needed (**Table 2**).

**Table 2:** Retrospective and Prospective Studies of Selectively Discontinuation of ICIs.

	Study	Author (Year)	Object	ICIs	No. of Patients discontinue therapy	Selectively discontinuation conditions	Median treatment duration (months)	PFS after discontinuation	OS after discontinuation	Retreatment response rate (CR+PR+SD)
**Melanoma**	KEYNOTE-001 (NCT01295827)	Robert (112) (2018)	Advanced melanoma	PEMB	67	Received PEMB for ≥ 6 months and at least two treatments beyond confirmed CR	23	24-month PFS 90%		3/3 (100%)
	KEYNOTE-006 (NCT01866319)	Robert (113) (2019)	Advanced melanoma	PEMB	23	Received PEMB for ≥ 6 months and at least two treatments beyond confirmed CR		24-month PFS 86%		1/1 (100%)
	NCT02673970	Jansen (114) (2019)	Advanced melanoma	anti-PD-1	185	Joint decision between patient and physician in the absence of PD or treatment limiting toxicity	12	24-month PFS 71%		11/19 (58%)
			*CR status at discontinuation*		*117*		*11*	*24-month PFS 86%*		*6/9 (67%)*
			*PR status at discontinuation*		*44*		*15*	*24-month PFS 68%*		*3/6 (50%)*
			*SD status at discontinuation*		*16*		*14*	*24-month PFS 50%*		*2/4 (50%)*
	Retrospective study	Zeijl (115) (2021)	Advanced melanoma	anti-PD-1	324	Joint decision between patient and physician in the absence of PD				17/27 (63%)
			*CR status at discontinuation*		*90*		*11.2*	*24-month PFS 64%*	*24-month OS 88%*	*5/6 (83%)*
			*PR status at discontinuation*		*190*		*11.5*	*24-month PFS 53%*	*24-month OS 82%*	*10/16 (63%)*
			*SD status at discontinuation*		*44*		*7.2*	*24-month PFS 31%*	*24-month OS 64%*	*2/5 (40%)*
	Retrospective study	Pokorny (116) (2021)	Advanced melanoma	anti-PD-1	52	Joint decision at 1 year (>6 months and <18 months) in the setting of ongoing treatment response or disease stability	11.1	median PFS 3.9m^a^		8/8 (100%)
	Retrospective study	Warner (117) (2020)	Advanced melanoma	anti–PD-1	102	Achieve CR	9.4	36-month PFS 72%	36-month OS 83%	41/78 (53%)
**NSCLC**	CheckMate 153 (NCT02066636)	Waterhouse (118) (2020)	Advanced NSCLC	NIVO		Receiving treatment and randomly assigned at end of 1 year				
			Continuous		89		25.6	12-month PFS 65% 24-month PFS 52%	12-month OS 86% 24-month 73%	
			*CR/PR status at random*		*62*			*median PFS 31.0m*	*NR*	
			*SD status at random*		*27*			*median PFS 11.8m*	*median OS 32.2m*	
			1-year fixed-duration		85		12	12-month PFS 44% 24-month PFS 31%	12-month OS 82% 24-month OS 61%	4/39 (10%)
			*CR/PR status at random*		*58*			*median PFS 10.6m*	*median OS 33.5m*	
			*SD status at random*		*27*			*median PFS 9.4m*	*median OS 26.6m*	
	KCSG LU20-11 Retrospective study	Kim (119) (2022)	Advanced NSCLC	anti–PD-1/L1		Completed 2 years of ICI therapy or were treated for more than 6 months and then discontinued ICIs without disease progression				
			Completed 2-years ICIs		96		24.0	12-month PFS 81%	12-month OS 96%	
			*CR/PR status at discontinuation*		*82*		*24.0*	*12-month PFS 84%*	*12-month OS 97.4%*	
			*SD status at discontinuation*		*14*		*24.0*	*12-month PFS 63.0%*	*12-month OS 90%*	
			Discontinued ICIs Early		43		10.5	12-month PFS 71%	12-month OS 90%	
			*CR/PR status at discontinuation*		*39*		*14.0/10.0*	*12-month PFS 80%*		
			*SD status at discontinuation*		*4*		*18.3*	*12-month PFS 50%*		
	INTEPI Retrospective study	Bilger (120) (2021)	Advanced NSCLC		54	Tumor controlled after at least 18 months of treatment	26	12-month PFS 71% 24-month PFS 63%	12-month OS 90% 24-month OS 84%	7/7 (100%)
			*CR/PR status at discontinuation*		*48*			*12-month PFS 76%*		
			*SD status at discontinuation*		*6*			*12-month PFS 22%*		
**Multiple Cancer Types**	Observational study	Abraham (121) (2022)	All cancers received at least one cycle of ICI	ICIs	1011	Real-world multicentric Indian data predominantly with short-course ICI therapy	3	median PFS 6.4m 12-month PFS 36% 24-month PFS 24%	median OS 13.6m 12-month OS 52% 24-month OS 35%	(<1%)
	Retrospective study	Iivanainen (122) (2018)	Metastatic cancer	anti-PD-1	17	Reaching the maximal restricted PD-1 therapy length (6 months)	4	median PFS 14m	median OS 27m	0/3 (0%)

ICIs, immune checkpoint inhibitors; PFS, progression-free survival; OS, overall survival; NIVO, nivolumab; PEMB, pembrolizumab; IPI, ipilimumab; CR, complete remission; PR, partial response; SD, stable disease; PD, progress disease; NR, not reached; NSCLC, non-small-cell lung cancer. The italicized parts represent subgroups.

a. After a median follow-up of 20.5 months from treatment discontinuation, the PFS rate was 75%.

### 4.1 Melanoma

Melanoma is the most effective and well-studied tumor in immunotherapy, and a series of published studies on selectively discontinuation of ICIs have focused on melanoma. In the revised protocol of the KEYNOTE-001, 006 studies, patients who received pembrolizumab for more than 6 months, whose tumors reached CR and followed by at least two cycles of pembrolizumab could choose to discontinue treatment early. A total of 67 and 23 CR patients discontinued treatment, and their 2-year PFS were 89.9% and 86.4%, which were similar to the outcomes of other enrolled patients (112, 113). A Belgium real-world cohort study analyzed 185 melanoma patients who chose to discontinue anti-PD-1 therapy in the absence of disease progression or toxicity, with a median treatment duration of 12 months and 1- and 2-year PFS after discontinuation of 90% and 71% (114). The risk of recurrence was significantly lower in CR patients than in partial response (PR)/stable disease (SD) patients. In CR patients, the risk of relapse was significantly higher for treatment duration of less than 6 months than for treatment duration of more than 6 months, but did not differ between 6-12, 12-18, 18-24 or more than 24 months (114). Another Netherlands observational study reviewed 324 patients who discontinued first-line anti-PD-1 monotherapy without disease progression and found a better outcome in patients with CR/PR status compared to patients with SD. The 2-year PFS and OS were 64% and 88%, 53% and 82%, 31% and 64% for CR, PR, and SD patients, respectively (115). Pokorny reviewed 52 metastatic melanoma who responded well to 1-year treatment and selectively discontinue therapy, with a median follow-up of 20.5 months after discontinuation 39 (75%) patients had no disease progression (116). A study from Memorial Sloan Kettering Hospital showed that CR patients discontinued therapy after a median treatment duration of 9.4 months, with a 3-year PFS of 72.1% and a 3-year OS rate of 82.7% (117). To sum up, a large proportion of melanoma patients who achieved CR could get sustained efficacy after ICIs discontinuation, with a low recurrence rate and a 2-year PFS of 65-85%. Therefore, there is a premature suggestion for melanoma that early discontinuation of ICIs could be considered for CR patients with 6-months additional treatment after achieving CR.

### 4.2 NSCLC

The best treatment response for most NSCLC patients is PR/SD rather than CR, and the optimal treatment duration may vary from melanoma. The CheckMate 153 study is the first phase III randomized controlled study of ICIs duration to explore whether continuation treatment provides a survival benefit for advanced NSCLC who are progression-free after 1-year nivolumab treatment. The results showed that patients who discontinued nivolumab had an increased risk of relapse and that continued nivolumab could provide a survival benefit. Further stratified analysis showed that CR/PR patients is benefit from continuous treatment, while for SD patients, the median PFS of continuous treatment and 1-year fixed treatment was similar, suggesting that continued treatment was more meaningful for CR/PR patients (118). A multicenter retrospective study from Korea reported the long-term follow-up results in patients with advanced and/or metastatic NSCLC (119). For patients who completed 2-years ICIs therapy, the 1-year PFS and OS were 81.1% and 96.4%, respectively. And for patients who discontinued ICIs after more than 6 months of treatment without disease progression, the 1-year PFS and OS were 71.0% and 90.0%, respectively. Among them, the risk of relapse was significantly higher for treatment duration of 6-12 months but did not differ between 12-18 or 18-24 months (119). Bilger reported that NSCLC patients who chose to discontinue the drug after at least 18 months of ICIs therapy had 1-year PFS and OS of 71% and 90%, and 2-year PFS and OS of 63% and 84%, respectively (120). Comparing the results of the above studies, the duration of treatment for ICIs was set at >6 months, 1 year, >18 months, 2 years, and continued until progression or intolerability. The numerical trend of the longer the treatment duration the lower the risk of recurrence, independent of the patient’s best response status. Therefore, we believe that the duration of ICIs for NSCLC may be longer than for melanoma, with a more reasonable cut-off of 18-24 months.

### 4.3 Multiple Cancer Types

A multicenter observational study in India found that a short course of ICIs (0.5-13 months) was comparable in clinical benefit rate to standard ICIs therapy from literatures (121). Oulu University Hospital restricted maximal PD-(L)1 therapy length to 6 months, and reviewed 17 responders discontinued PD-1 therapy after 6 months therapy, 11 of whom remained stable after 1 year (122).

## 5 Ongoing Clinical Trials Related to ICIs Regimen Optimization

A randomized clinical trial initiated by the University of Chicago sought to compare the standard interval and extended interval dosing of nivolumab (240mg Q2W/480mg Q4W vs 240mg Q4W/480mg Q8W) or pembrolizumab (200mg Q3W/400mg Q6W vs 200mg Q6W) in locally advanced or metastatic cancers (123). Roswell Park Cancer Institute sponsored another multicenter randomized trial compared the regimen of pembrolizumab 200mg Q3W with 200mg Q12W in NSCLC patients benefit from pembrolizumab monotherapy, aiming to reduce the dose intensity of pembrolizumab in the maintenance phase (124). There is also a dose tapering and early discontinuation trial for NSCLC initiated by Radboud University Medical Center, where the labelled dose of pembrolizumab (200mg Q3W/400mg Q6W) will be reduced to 300mg Q6W (125). The ADAPT-IT study enrolled advanced melanoma underwent CT scan after 2 cycles of nivolumab combined with ipilimumab. According to CT results, patients with early favorable antitumor effect discontinued the combination and transferred to nivolumab monotherapy, otherwise received 4 cycles of combination therapy. Interim results were reported that 41/60 patients (68%) experienced only 2 cycles of combination, with 12-months PFS and OS of 68% and 85%, 18-months PFS and OS of 52% and 80%, respectively (126). Patients with early favorable antitumor effect who received additional combination therapy did not significantly improve their outcomes. There are also some small-sample studies focusing on reduced doses of ICIs combined with radiotherapy, chemotherapy, etc., and the use of artificial intelligence to guide medication (127–129).

A number of clinical trials focusing on ICIs discontinuation are carrying out. Similar to CheckMate 153, the Japanese phase III SAVE study (130)recruits NSCLCs with good response and no serious side effects after 1-year anti-PD-1/L1 therapy, the French DICIPLE study (131) recruits patients with stage IV NSCLC without progression after 6-months combination therapy of nivolumab plus ipilimumab, and the UK phase III DANTE study (132) recruits progression-free melanomas after 1-year anti-PD-1 therapy. The patients were randomized into the continuation and discontinuation groups to compare the survival differences. There are also some clinical trials exploring the modes of determining the treatment duration based on treatment response rather than setting a fixed duration, e.g., the Netherlands Safe Stop study (133) hopes to answer the question of whether ICIs can be discontinued after achieving CR/PR in melanoma. Moreover, many clinical studies exploring intermittent treatment patterns. For instance, Canadian STOP-GAP study (134) evaluates the clinical feasibility of stopping treatment after maximal tumor response and retreating when disease progresses. In another intermittent dosing mode, patients were evaluated periodically, and treatment was discontinued if tumors decreased by 10% or more, continued if tumors did not decrease, restarted in patients with a ≥ 10% tumor increase and again held with tumor reduction ≥10%. Although clinical trial was closed prior due to changes in standard of treatment, still providing experience for further investigation of intermittent immunotherapy dosing strategies (135). TITAN-RCC/TCC trial explored a response-based tailored immunotherapy approach, starting with nivolumab induction, followed by nivolumab monotherapy maintenance in responders, boost combination therapy of nivolumab plus ipilimumab in non-responders (136, 137). It is also important to find accurate markers for discontinuation and methods of monitoring after discontinuation. In a retrospective study at Georgetown Lombardi Cancer Center, inactive melanoma confirmed by PET/CT or tumor biopsy had a relapse rate of less than 10% at one year after anti-PD-1 withdrawal (138). Inspired by this, they initiated a clinical trial in which melanoma patients underwent PET-CT scan after 1-year ICIs treatment, and the negative PET-CT results guided drug discontinuation (138). We look forward to the results of all the above studies (**Table 3**), meanwhile, it is worth noting that most clinical studies are mainly limited to melanoma and NSCLC, and it is uncertain whether the results can be directly extrapolated to other tumor types; more prospective studies with predictive biomarkers are needed.

**Table 3:** Ongoing clinical trials related to ICIs regimen optimization. (Comparing the modified dosing regimens with the standard dosing regimens).

Study	ICIs	Sponsor	Objective	Study Type	Estimated Enrollment	Dosing Regimens	Primary Endpoints	Secondary Endpoints	Status	Estimated Study Completion Dates
**Dose Reduction**										
NCT04295863	NIVO/PEMB	University of Chicago (123)	locally advanced or metastatic cancers	Randomized Open Label	264	NIVO Standard: 240mg Q2W/480mg Q4W vs NIVO Extended: 240mg Q4W/480mg Q8W PEMB Standard: 200mg Q3W/400mg Q6W vs PEMB Extended: 200mg Q6W	noninferiority	efficacy	Recruiting	April, 2025
NCT04032418	PEMB	Roswell Park Cancer Institute (124)	NSCLC	Randomized Open Label	152	PEMB 200mg Q3W Vs PEMB 200mg Q12W	1-year PFS	OS, irAEs	Recruiting	September, 2023
DEDICATION-1 NCT04909684	PEMB	Radboud University Medical Center (125)	NSCLC	Randomized Open Label	750	PEMB 200mg Q3W/400mg Q6W Vs PEMB 300mg Q6W	1-year OS	median OS, 2-year OS, best overall response, DCR, duration of treatment, QOL	Recruiting	November, 2024
ADAPT-IT NCT03122522	IPI+NIVO	Memorial Sloan Kettering Cancer Center (126)	unresectable III or stage IV metastatic melanoma	Single Group Assignment Open Label	70	2 doses of the N1I3^a^+ NIVO maintenance or 4 doses of the N1I3^a^+ NIVO maintenance	objective response rate	NA	Active, not recruiting	April, 2023
NiCOL NCT03298893	NIVO	Institut Curie (127)	locally advanced cervical cancer	Single Group Assignment Open Label	21	5 weeks of radiochemotherapy + NIVO followed by 5 months of NIVO alone NIVO doses: flat dose 240 mg Q2W vs 1mg/kg Q2W	occurrence of DLTs	ORR, PFS, DFS circulating tumor DNA heterogeneity	Completed	March, 2022
NCT04817254	NIVO+IPI	National Cancer Institute (128)	Glioblastoma/Gliosarcoma	Randomized Open Label	48	NIVO + IPI 1mg/kg + TMZ vs NIVO + IPI 3mg/kg + TMZ	OS	T cell response	Recruiting	December, 2024
NCT05175235	NIVO	National University Hospital, Singapore (129)	solid tumors treatment with NIVO for 12 months	Single Group Assignment Open Label	10	Using artificial intelligence technology provide dynamic dose optimization throughout treatment.	CURATE.AI^b^ applicability	adherence clinically significant dose changes	Recruiting	August, 2023
**Selectively Discontinuation**										
SAVE study jRCT1031190032	ICIs	National Cancer Center Research and Development Fund of Japan (130)	Advanced NSCLC responded well to ICIs	Randomized Open Label	216	Patients who have responded well to PD-1 pathway inhibitors for >12 months Continue treatment vs Discontinue treatment	OS	PFS, time to treatment failure of strategy, response rate, PFS after resuming ICIs, safety	Recruiting	NA
DICIPLE NCT03469960	NIVO+IPI	Intergroupe Francophone de Cancerologie Thoracique (131)	Stage IV NSCLCs responded well to NIVO+IPI	Randomized Open Label	265	Patients who are progression-free after 6-months NIVO 3mg/kg Q2W+IPI 1mg/kg Q6W Continue NIVO+IPI treatment vs Discontinue treatment	PFS	QOL, OS, biological correlative exploratory studies	Active, not recruiting	May, 2023
DANTE ISRCTN15837212	ICIs	Sheffield Teaching Hospitals NHS Trust (132)	melanomas with progression-free after 1-year anti-PD-1 therapy	Randomised controlled trial	1208	Patients who are progression-free after 1-year ICIs therapy Continue treatment vs Discontinue treatment	PFS	QOL, OS, ORR, Best tumor response rate, DOR, Safety and toxicity, Cost-effectiveness	Recruiting	May, 2027
Safe Stop NL7293	NIVO/PEMB	Erasmus Medical Centre Fellowship grant (133)	advanced and metastatic melanoma achieving CR/PR	Single Group Assignment Open Label	200	Patients with a confirmed CR/PR discontinue treatment, continue scans according to standard of care	the rate of ongoing responses at 24 months after discontinuation	best overall and duration of response, need and outcome of rechallenge with PD-1 blockade, safety, QOL	Active, not recruiting	NA
STOP-GAP study NCT02821013	Anti-PD-1	Canadian Cancer Trials Group (134)	metastatic melanoma	Randomized Open Label	614	Intermittently treatment vs Continuously treatment	OS	PFS, response rate, DOR, irAEs, QOL, economic evaluation	Recruiting	December, 2029
NCT03126331	NIVO ± IPI	Case Comprehensive Cancer Center (135)	renal cell carcinoma	Single Group Assignment Open Label	26	Discontinue treatment if tumor decreases by 10% or more, continue treatment if tumor is not decreased. After received NIVO+IPI*4 + 24 weeks NIVO, SD will continue with NIVO maintenance, PR/CR will discontinue.	feasibility of intermittent therapy	clinical outcome (ORR, PFS, OS) toxicity	Active, not recruiting	October, 2023
TITAN-RCC NCT02917772	NIVO/IPI	AIO-Studien-gGmbH (136)	Metastatic or Advanced Renal Cell Carcinoma	Single Group Assignment Open Label	200	Induction: NIVO 240mg Q2W *8 If CR/PR: NIVO Monotherapy Maintenance If SD/PD: Boost Combination Therapy (N3I1 *2-4)^c^	ORR	remission rates, time-to-response, time to immunotherapy resistance, DOR, PFS, OS, safety	Active, not recruiting	September, 2022
TITAN-TCC NCT03219775	NIVO/IPI	AIO-Studien-gGmbH (137)	advanced or metastatic transitional cell carcinoma	Single Group Assignment Open Label	169	Induction: NIVO 240mg Q2W *4 If CR/PR: NIVO Monotherapy Maintenance If SD/PD: Boost Combination Therapy (N1I3 *2-4)^a^	ORR	remission rates, time-to-response, time to immunotherapy resistance, DOR, PFS, OS, safety	Active, not recruiting	February, 2023
The PET-Stop NCT04462406	NIVO/PEMB/IPI	ECOG-ACRIN Cancer Research Group (138)	stage IIIB-IV melanoma	Sequential Assignment Open Label	150	Decision to early discontinue or continue treatment based on biomarkers seen on PET/CT imaging and tumor biopsy.	event free survival	Rates of pathologic response, OS, irAEs	Recruiting	August, 2026

ICIs, immune checkpoint inhibitors; NIVO, nivolumab; PEMB, pembrolizumab; IPI: ipilimumab; DLT, dose-limiting toxicity; ORR, objective response rates (CR + PR); PFS, progression-free survival; DFS, disease-free survival; NSCLC, non-small-cell lung cancer; OS, overall survival; irAEs, ICIs-related adverse effects; QOL, quality of life; DCR, disease control rate; CR, complete remission; PR, partial response; SD, stable disease; DOR, duration of response. *, multiple the number of cycles.

a. N1I3: Combination treatment with nivolumab 1 mg/kg q3w plus ipilimumab 3 mg/kg q3w.

b. CURATE.AI: An artificial intelligence-derived platform helps to find an appropriate dosing strategy developed by National University of Singapore.

c. N3I1: Combination treatment with nivolumab 3 mg/kg q3w plus ipilimumab 1 mg/kg q3w.

Previously established methodology of dose-finding in early-stage clinical trials has not progressed with the therapeutic improvements, and the concept of maximally tolerated doses (MTD) has much less instructive for ICIs recommended dose. If the MTD is not reached during dose escalation, the recommended phase 2 dose could be evaluated based on safety profile, pharmacokinetics/pharmacodynamics modelling simulations, early efficacy biomarkers, the variation of immunological composition reflecting immunomodulatory effect, target engagement receptor occupancy model and other new parameters (139–143). Besides, appropriate extension of the follow-up period may help to reduce the bias brought by unrecognized toxicity. It is a trend to design trials with reference to the minimum effective dose and supplementing it with *in silico* modelling and simulation, as well as incorporated TDM strategy to monitor trough concentrations within the therapeutic range, enable the provision of precision dosing of ICIs. Various novel phase I/II clinical trial designs have been proposed in the literature to select the optimal biological dose, such as Bayesian adaptive design (144–148). In terms of modified the current dosing schedule, the viewpoint published on JAMA ONCOLOGY proposed that Bayesian noninferiority studies could be more efficient to demonstrate the comparability between the modified regimen and the established standard one, especially when utilized some predictive biomarkers, pharmacokinetic, or pharmacodynamic end points (11). Besides, there is also support for noninferiority trials designed with relatively wide margins, considering the trade-off between statistical certainty, feasibility and population health (12).

## 6 Medication Strategies for ICIs Under the COVID-19 Pandemic

Cancer patients are in an immunosuppressed state and are at high risk for COVID-19 infection, with a high incidence of severe cases and mortality (149, 150). Due to the lack of evidence, experts are divided on the therapeutic management of ICIs in cancer patients during the COVID-19 pandemic. Some suggest that delayed or early discontinuation of therapy may be considered for elderly patients with comorbidities and low tumor burden, those who achieve (or near) CR, and those receiving adjuvant therapy (149, 151, 152). Others believe that the susceptibility and severity of cancer patients to COVID-19 may simply be an epidemiological coincidence caused by bias (153–155). Immunotherapy can restore the immune function, and patients receiving ICIs may be more resistant to the virus than those receiving chemotherapy and targeted therapy (156). The duration of the COVID-19 pandemic remains unpredictable and should not prevent the use of ICIs in patients with highly responsive tumors (157–159). The current consensus is that both physicians and patients preferred high-dose, extended-interval regimen to reduce the risk of exposure (73, 160–163). There was no difference in safety and efficacy between extended-interval dosing and standard dosing pembrolizumab or durvalumab in NSCLC patients during the COVID-19 pandemic, while PFS, OS were longer in patients treated with extended-interval nivolumab (163).

The safety and/or efficacy of the COVID-19 vaccine, as well as the interaction between the vaccine and the ICI, are inconclusive for patients treated with ICI whose immune systems are activated. The limited evidence available support COVID-19 vaccination in patients treated with ICIs. Chen YW (164) and Waissengrin (165) reported no serious vaccine-related adverse reactions observed in 81 and 137 patients receiving ICIs and COVID-19 mRNA vaccine, and no patients developed new irAEs or exacerbation of existing irAEs. The prospective VOICE study (vaccination against COVID in cancer), which included chemotherapy and immunotherapy patients, also confirmed the safety and efficacy of the mRNA-1273 vaccine (166). A recently published multicenter observational study included 2048 patients who had previously received anti-PD-1 therapy and were divided into vaccinated subgroup (receiving inactivated SARS-CoV-2 virus vaccine) and non-vaccinated subgroup for comparison. Both subgroups were similar in terms of ICIs efficacy, while in terms of safety, the vaccinated subgroup was more likely to have mild irAE, while the incidence of severe irAE was instead reduced (167). Although case of cytokine release syndrome occurring 5 days after BTN162b2 mRNA COVID-19 vaccination in patient with long-term anti-PD-1 therapy have been reported (168), the benefit-risk profile still strongly supports vaccination in cancer patients. The current recommendation is that patients with active cancer undergoing immunotherapy should receive COVID-19 vaccine at the earliest available opportunity but should avoid vaccination 48-72 hours within treatment to reduce confusion about the causality of adverse effects (169–172). The patients undergoing combined ICIs therapy should be more carefully evaluated and closely monitored at the time of vaccination (173, 174).

## 7 Conclusions

Rational dose selection and optimization of dosing regimens are of clinical importance and are prerequisites for enhancing patients’ medication compliance and obtaining maximum clinical benefits. The management of ICIs is still on research phase, and the approved dosing regimen may not be the best. The previously established approach of early-phase dose-finding clinical trials is not appropriate for current immunotherapy. Various phase I/II clinical trial designs have been proposed to select the optimal biological dose of ICIs, pending test of reasonableness in practice. There is an emergent need to explore the efficacy and safety of modified ICIs treatment strategies (e.g., lower dosage or shorter course) to promote the use of ICIs and reduce drug toxicity and economic wastage. Pharmacokinetic/pharmacodynamic studies, early clinical trials, and small sample attempts suggest that lower-dose and less-frequency administration of ICIs may have durable effects, similar to those of standard dosing regimens. Compared to reduce single-dose, the better way to reduce dose intensity is probably to extend the dosing frequency, which is more economical and convenient, especially in the context of COVID-19 pandemic. The search for the optimal duration of ICIs is also progressing, from a fixed course of treatment to determining the duration of treatment based on treatment response, and further searching for imaging and biological biomarkers to help determine the timing of drug discontinuation. It is important to note that the optimal dosing regimen of ICIs is related to the immunogenicity of the tumor, disease stage, and physical status of patients, and extrapolation of results requires caution. In the era of precision medicine, we pursue individualized treatment rather than using the same schedule for all patients. More pharmacokinetic/pharmacodynamic studies, interventional pharmacoeconomics clinical trials and real-world data, as well as in-depth studies on the mechanisms of ICIs are very essential. These off-labelled dose de-escalation of ICIs in clinical practice would be under the guidance and collaboration of pharmaceutical manufacturers. The correlated research potentially of interest to insurers, government payers, academic institutions, as well as professional/patient associations. Although the study funding and the dissemination of the concepts may be difficult, it is of great interest and urgently needed to reduce the medical stress on both individuals and society.

## Author Contributions

MJ contributed to the study conception and design, data acquisition, data analysis and interpretation, manuscript writing and revision. MJ, YH and GL contributed to the study data acquisition, data analysis and interpretation, original draft preparation. MJ and CC contributed to review and revise the manuscript. All authors agree to be accountable for the content of the work.

## Conflict of Interest

The authors declare that the research was conducted in the absence of any commercial or financial relationships that could be construed as a potential conflict of interest.

## Publisher’s Note

All claims expressed in this article are solely those of the authors and do not necessarily represent those of their affiliated organizations, or those of the publisher, the editors and the reviewers. Any product that may be evaluated in this article, or claim that may be made by its manufacturer, is not guaranteed or endorsed by the publisher.
